# Separation, Identification, and Antidiabetic Activity of Catechin Isolated from *Arbutus unedo* L. Plant Roots

**DOI:** 10.3390/plants7020031

**Published:** 2018-04-12

**Authors:** Hanae Naceiri Mrabti, Nidal Jaradat, Ismail Fichtali, Wessal Ouedrhiri, Shehdeh Jodeh, Samar Ayesh, Yahia Cherrah, My El Abbes Faouzi

**Affiliations:** 1 Faculty of Medicine and Pharmacy, Laboratory of Pharmacology and Toxicology, Pharmacokinetics Team, Mohammed V University in Rabat, Rabat Institute, Rabat BP 6203, Morocco; y.cherrah1@um5s.net.ma (Y.C.);myafaouzi8@yahoo.fr (M.E.A.F.); 2 Department of Pharmacy, Faculty of Medicine and Health Sciences, An-Najah National University, P.O. Box 7, 00970 Nablus, Palestine; 3 Laboratory of Applied Organic Chemistry, Faculty of Science and Technology, Sidi Mohamed Ben Abdellah University, Immouzer Road, 30050 Fez, Morocco; biocmb@gmail.com; 4 Laboratory of Medicinal and Aromatic Plants and Natural Substances, National Institute of Medicinal and Aromatic Plants-Taounate, Sidi Mohamed Ben Abdellah University, 30050 Fez, Morocco; wessal.ouedrhiri@gmail.com; 5 Department of Chemistry, An-Najah National University, P.O. Box 7, 00970 Nablus, Palestine; Sjodeh@hotmail.com; 6 Physical Science Department, Harold Washington College, 10 E. Lake Street, Chicago, IL 60601, USA; sayesh@ccc.edu

**Keywords:** *Arbutus unedo* L., α-glucosidase, catechin, HPLC, NMR

## Abstract

Phytopharmaceuticals play an essential role in medicine, since the need to investigate highly effective and safe drugs for the treatment of diabetes mellitus disease remains a significant challenge for modern medicine. *Arbutus unedo* L. root has various therapeutic properties, and has been used widely in the traditional medicine as an antidiabetic agent. The current study aimed to isolate the pharmacologically active compound from *A. unedo* roots using accelerated solvent extraction technology, to determine its chemical structure using different instrumental analytical methods, and also to evaluate the α-glucosidase inhibitory activity. The roots of *A. unedo* were exhaustively extracted by high-pressure static extraction using the Zippertex^®^ technology (Dionex-ASE, Paris, France), and the extract was mixed with XAD-16 resin to reach quantifiable amounts of active compounds which were identified by high-pressure liquid chromatography (HPLC), ^1^H NMR (300 MHz), and ^13^C NMR. The antidiabetic activity of the isolated compound was evaluated using the α-glucosidase inhibitory assay. The active compound was isolated, and its structure was identified as catechin using instrumental analysis.The results revealed that the isolated compound has potential α-glucosidase inhibitory activity with an IC_50_ value of 87.55 ± 2.23 μg/mL greater than acarbose. This was used as a positive control, which has an IC_50_ value of 199.53 ± 1.12 μg/mL. According to the results achieved, the roots of *A. unedo* were considered the best source of catechin and the Zippertex^®^ technology method of extraction is the best method for isolation of this therapeutic active compound. In addition, the α-glucosidase inhibitory activity results confirmed the traditional use of *A. unedo* roots as an antidiabetic agent. Future clinical trials and investigations of antidiabetic and other pharmacological effects such as anticancer are required.

## 1. Introduction

Diabetes mellitus is a disorder of carbohydrate metabolism characterized by the impaired ability of the body to produce or respond to insulin, and thereby maintain proper levels of glucose in the blood. This produces several devastating effects and complications, including neuropathy, nephropathy, retinopathy, hyperthyroidism, hypertension, arteriosclerosis, and many other serious diseases [1,2,3]. In fact, other complications are associated with the used antidiabetic drugs, as regular administration can lead to several adverse effects [4]. Accordingly, the achievement of sufficient control of hyperglycemia is difficult to reach with commercially available antidiabetic medications, thereby resulting in various and serious complications [5]. In fact, the investigation on medicinal plants usually started with extraction procedures which play a crucial role in the extraction outcomes, e.g., yields percentages and the quality of the produced phytochemicals. Nowadays, a wide range of technologies with different methods of extraction is available. Zippertex technology is one of the most efficient methods of extraction. It is a high-pressure static extractor which is considered the most efficient and convenient solid/liquid extraction device. It combines extraction and filtration steps, offering limpid highly concentrated extracts, ready for chemical and biological investigations. The combination of static pressure and heating favor the access of the solvent into the heart of the solid matter, and increases the solubilization of the target compounds. Zippertex offers the maximum qualitative and quantitative recovery, with the minimum required operations, solvents, handling, and time [6,7].

Medicinal plants offer a great opportunity to discover new natural therapeutic molecules. Some of these molecules may have beneficial effects on glucose homeostasis in diabetic patients without causing any undesirable effects currently observed in modern antidiabetic agents [5,8].

*Arbutus unedo* L. (Ericaceae family) is commonly known as wild strawberry, which is a perennial small tree native to the Mediterranean basin countries. It constitutes an important contribution to the nutritional culture and to the health promotion of Moroccan community [9,10].

However, in traditional medicine of many Mediterranean countries, *A. unedo* plant has been used widely with the employment of decoctions and infusions of all plant parts: fruits, leaves, barks, and roots [11,12]. For instance, the fruits are well known in folk medicine as an antiseptic, laxative, and diuretic [13,14], while the leaves are used as an astringent, urinary antiseptic, diuretic, antidiarrhea, and depurative. Moreover, recently, the leaves have been used to treat inflammatory diseases, hypertension, and diabetes [11].

Furthermore, *A. unedo* roots havebeen used traditionally to treat various gastrointestinal, urological, dermatologic, and cardiovascular diseases, as well as a diuretic, anti-inflammatory, and antidiabetic agent [15,16,17,18].

In fact, the *A. unedo* extract allowed us to identify several familial compounds, such as terpenoids, free quinine, and anthraquinone, which were the subject of our previous work and others, such as polyphenols, flavonoids, and tannins, which were quantified in the plant roots aqueous extract, thus, its antioxidant activity was also evaluated [10].

The current study aimed to isolate the therapeutic active molecule of *A. unedo* roots using accelerated solvent extraction Zippertex technology with XAD-16 resin, characterize its structure using different spectra methods and to evaluate its α-glucosidase inhibitory activity.

## 2. Results

**Figure 1:**
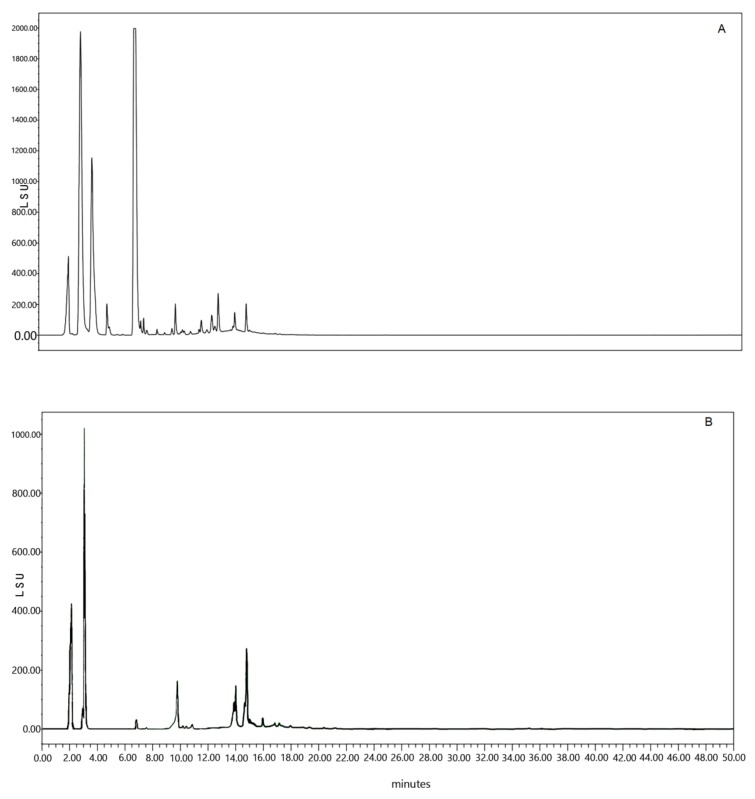
(**A**) HPLC analysis of *Arbutus unedo* roots aqueous extract obtained by Zippertex apparatus. (**B**) The aqueous extract of *A. unedo* roots adsorbed by the amberlite XAD-16.

The results of the present investigation showed that the extraction of *A. unedo* roots utilizing the Dionex-ASE (accelerated solvent extraction) prototype Zippertex produced a high percent of aqueous extract yield with 23.7%. The HPLC chromatogram of *A. unedo* rootsaqueous extract showed that this extract contained several phytochemical compounds, as shown in Figure 1A. This makes it difficult to identify the pharmacologically active principle, which encouraged us to look for another experimental method of separation, from which we can isolate a single therapeutic molecule. Satisfactory results were obtained using the XAD-16 resin. After adsorption on XAD-16 resin, the expected compound from the extract was separated using semi-preparative HPLC technique, and its chemical structure was identified as shown in Figure 1B.

**Figure 2:**
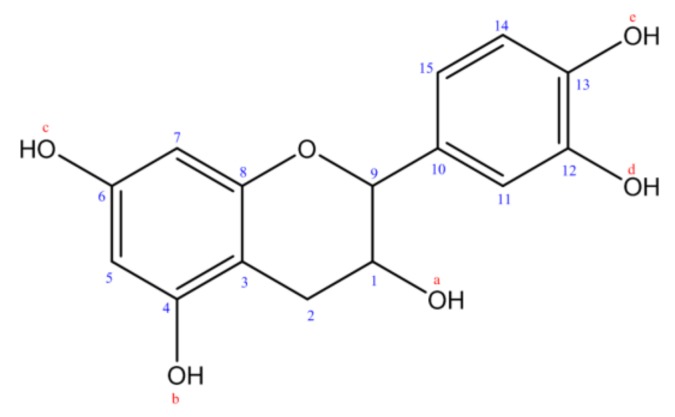
Catechin chemical structure isolated from *A. unedo* roots using XAD-16 resin.

The isolated compound showed interesting α-glucosidase inhibitory activity, results as shown in Table 1, in comparison with commercially used α-glucosidase inhibitory drug acarbose. These results encouraged us to identify and characterize the structure of this compound (Figure 2) using high-resolution mass spectrometry and NMR (^1^H and ^13^C) analyses. The negative-ion HRESIMS analysis gave the molecular formula C_15_H_14_O_6_ on the basis of *m*/*z* 289.10 [M − H]^−^ (calculated for C_15_H_13_O_6_) containing nine unsaturation.

The ^1^H and ^13^C NMR spectra show the following signals:

^1^H NMR (MeOD, 300 MHz): 2.46–2.54 (m, 1H, CH_2(2)_); 2.81–2.88 (m, 1H, CH_2(2)_); 3.29 (s, 1H, OH_(a)_); 3.97 (dd, 1H, CH–OH_(1)_, *J* = 7.5 Hz, *J* = 2.4 Hz); 4.57 (d, 1H, O–CH_(9)_, *J* = 7.5 Hz); 4.85 (s, 4H, 4OH_(b,c,d,e)_); 5.86 (d, 1H, CH_(5)_, *J* = 2.4 Hz); 5.93 (d, 1H, CH_(7)_, *J* = 2.1 Hz); 6.69–6.78 (m, 2H, 2CH_(14,15)_); 6.84 (d, 1H, CH_(11)_, *J* = 1.8 Hz).

^13^C NMR (MeOD, 75 MHz): 27.09 (CH_2(2)_); 67.40 (CH_(1)_); 81.43 (CH_(9)_); 94.17 (CH_(7)_); 94.96 (CH_(5)_); 99.49 (C_(3)_); 113.89 (CH_(11)_); 114.76 (CH_(14)_); 118.69 (CH_(15)_); 130.82 (C_(10)_); 144.81 (C_(13)_); 144.84 (C_(12)_); 155.51 (C_(8)_); 156.15 (C_(4)_); 156.39 (C_(6)_).

The ^1^H and ^13^C NMR spectra are shown in the Supplementary Material (Figures S1–S6).

The α-glucosidase inhibitory assay results showed that catechin inhibited α-glucosidase enzyme more than two folds of acarbose with IC_50_ value 87.55 ± 2.23 μg/mL against 199.53 ± 1.12 μg/mL of acarbose, as represented in Table 1.

## 3. Discussion

Due to problems associated with high processing temperatures and long processing times in conventional extraction methods, which can degrade or undergo undesirable oxidation processes, there is an essential need to promote development and application of alternative extraction techniques for phenolic compounds. Accelerated solvent extraction is a promising eco-friendly alternative providing exceptional separation and protection from degradation of unstable polyphenols. The current investigation revealed that extraction method of *A. unedo* roots utilizing of the Dionex-ASE (accelerated solvent extraction) prototype Zippertex produced a high yield of aqueous extract. Several conducted studies showed that the modern techniques, which have been replacing conventional ones, include accelerated solvent extraction, microwave-assisted extraction, supercritical fluid extraction, pressurized liquid extraction, and ultrasound-assisted extraction. These alternative techniques increased the extraction yields of polyphenols, reduced considerably the use of solvents, and accelerated the extraction process [19,20,21].

Flavonoid is a class of phytogenic polyphenolic compounds, including in many kinds of human diet, and has various physicochemical characters and chemical structures. The high-resolution mass spectrometry and NMR results revealed the presence of catechin in the roots of *A. unedo* extract, and these results are similar to those which were conducted by Junior et al. [22].

In fact, *A. unedo* fruits are considered as an alternative source of flavan-3-ols, in particular, catechin and its derivatives [2]. However, the inhibitory effect of catechin against α-glucosidase enzyme was evaluated in several previously conducted studies which initially demonstrated that catechin preferentially inhibited maltase rather than sucrase in an immobilized α-glucosidase inhibitory assay system [23,24,25,26]. This suggests that the α-glucosidase inhibition induced by catechin is closely associated with the presence of a free hydroxylgroupat the 3-position of this molecule [23]. Catechin is a very common and widely diffused metabolite in the plant kingdom. The traditional use of *A. unedo* as antidiabetic agent may be associated with other phytoconstituents, because many plants contain catechin. Therefore, the elevated biological property of this plant is due to the whole phytocomplex, and not only to just one molecule [27,28].

In addition, several conducted studies pointed out the interest of using catechin for many of health benefits, such as anticancer, antifungal, antioxidant, and antidiabetic purposes [29,30].

However, in a study established by Albuquerque et al. [21], they attempted the optimization of extraction of this molecule using different extraction methods, such as maceration and microwave techniques, and were capable of yielding 1.38 ± 0.1 and 1.70 ± 0.3 mg/g DW of catechin, respectively using the optimal extraction conditions. These extraction methods were found to be the most effective methods. The catechin yield from *A. unedo* root in the present study was of 95 mg of the plant dry weight. This high yield, which was obtained by using the Dionex-ASE (accelerated solvent extraction) prototype, Zippertex, in addition to the XAD-16 resin, which was used as an adequate option for an efficient cleanup step for purification and isolation of pure catechin compound from the plant root extract [31]. This yield was considered high in comparison with previously conducted extraction methods [21]. Moreover, the roots of *A. unedo* was considered the best source for isolation of the pharmacologically active molecule catechin in comparison with previously conducted isolation procedures of catechin from different sources [21]. However, many conducted research studies have demonstrated that the Zippertex method is friendly to the environment, with low cost and a low requirement for solvents, which decreased the time of extraction procedure and combined extraction/filtration steps [23].

The inhibitory effect of catechin against the α-glucosidase enzyme has been documented before; however, the advanced purification method used, with a greater percentage of yield, is novel.

To the best of the authors’ knowledge, the current study is the first one which was carried out with the intent of isolation of the therapeutically active compound from the *A. unedo* plant roots, and to find out the best method for isolation of catechin from this plant. Moreover, this investigation approved the traditional antidiabetic use of *A. unedo* plant roots. Furthermore, in vivo trials are required to support this therapeutic use and to design a suitable dosage form to produce new drugs or supplements from *A. unedo* plant roots extract, to help in reducing blood glucose level and its complications.

## 4. Materials and Methods

### 4.1. Plant Material

The *A. unedo* roots were collected at Beni Mellal region, Morocco, in October 2016. The voucher specimen has been deposited in the Herbarium of the Botany Department at the Scientific Institute of Rabat/Morocco and then voucher specimen code is (RAB 101548). The roots were naturally dried in the shade at room temperature for 2–3 weeks.

### 4.2. Extraction and Isolation Procedures

The dry roots of *A. unedo* plantwere mechanically powdered, and 30 g of the plant material was extracted with 200 mL of water at 100 °C under static nitrogen pressure (100 bars) using the Dionex-ASE (accelerated solvent extraction) prototype Zippertex. The obtained aqueous extract was evaporated using a Rotavap at 100 °C, and lyophilized to give a brown powder which produced 7.12 g of yield (23.7%). After that, 1 g of the obtained extract was adsorbed by the amberlite XAD-16 (Sigma, Steinheim, Germany), which was previously washed with methanol. The pharmacological active molecule catechin was isolated from roots of *A. unedo* by a methanol-resin reagent, and the yield was 95 mg with 1.33% of the total aqueous extract [32].

### 4.3. Analytical Methods

#### 4.3.1. Analytical HPLC

An Alliance^®^ Waters W2695 HPLC chain (Waters Corporation, Milford, MA, USA) equipped with a Waters 2996 PDA detector (Waters Corporation, Milford, MA, USA) equipped with a Sunfire III C18 (4.6 mm × 150 mm) 3.5 μm (Waters) reverse phase column. This chromatographic system is coupled to a Waters 2424 light scattering (DEDL) detector. The HPLC system is controlled by Empower 3 software (Waters) (Waters Corporation, Milford, MA, USA). While ultrapure water (MilliQ), 0.1% formic acid/acetonitrile and 0.1% formic acid were the used solvents. In addition, the gradient was from 0 to 100% acetonitrile in 40 min and 10 min to 100% acetonitrile with flow rate of 0.7 mL/min [7].

#### 4.3.2. HPLC Semi-Preparative

The pharmacological active molecule of *A. undo* roots was isolated by a methanol-resin reagent (95 mg) was identified by preparative HPLC chain equipped with a Waters 717 auto-sampler, a Waters 600 pump, a Waters 2998 DEDL, Waters 2420, PDA detector, and a Waters AF degasser. The column used is a Sunfire III C18 (10 mm× 250 mm) 5 μm. The HPLC system is controlled by Empower 2 software (Waters) (Waters Corporation, Milford, MA, USA). While ultrapure water (MilliQ), 0.1% formic acid/acetonitrile and 0.1% formic acid were the used solvents with a flow rate of 4 mL/min [7].

#### 4.3.3. NMR and HRMS Analysis

The^1^H NMR (300 MHz) and ^13^C NMR (75 MHz) spectra were recorded on Bruker spectrometer with chemical shift values (δ) given in part per million (ppm) relative to TMS (0.00 ppm) and using MeOD as a solvent, the coupling constants (J) are expressed in hertz (Hz) and singlet (s), doublet (d), and doublet of a doublet (dd) as well as the multiplet (m). The high-resolution mass spectra (HRMS) analysis was performed in a negative mode in full mass scan (*m/z* 100 to 600 amu) using a Thermo Scientific Orbitrap Mass Spectrometer Exactive equipped with a heated electrospray ionization source (HESI), and the used resolution was 1000 [22,33].

### 4.4. Enzyme Inhibition Assay

The α-glucosidase inhibition assay was performed according to the slightly modified method of Kee et al. [34], with some modifications. Briefly, α-glucosidase enzyme (0.1 U/mL) (Sigma-Aldrich, Lyon, France) and substrate *p*-nitrophenyl-α-d-glucopyranoside (*p*-NPG, 1 mM) (Sigma-Aldrich, Lyon, France) were dissolved in potassium phosphate buffer (0.1 M, pH 6.7), and all samples were dissolved in distilled water. The inhibitor (150 μL) was pre-incubated with the enzyme (100 μL) at 37 °C for 10 min, then a substrate (200 μL) was added to the reaction mixture. The enzymatic reaction was performed at 37 °C for 30 min. The reaction was then terminated by the addition of Na_2_CO_3_ (1 M, 1 mL) (Sigma-Aldrich, Lyon, France). All the samples were analyzed in triplicate with different concentrations to determine the IC_50_ values, and the absorbance was recorded at 405 nm. The α-glucosidase inhibitory activity was expressed as the percentage of inhibition, and the IC_50_ values were determined. Acarbose (Sigma-Aldrich, Lyon, France) was used as the positive control. The results were expressed as percentage inhibition and calculated using the following equation:

$$\mathrm{Inhibition}\;\left(\%\right)=\frac{\left(\mathrm{Ac}-\mathrm{Acb}\right)-\left(\mathrm{As}-\mathrm{Asb}\right)}{\mathrm{Ac}-\mathrm{Acb}}\times 100$$

where Ac refers to the absorbance of the control (enzyme and buffer), Acb refers to the absorbance of the control blank (buffer without enzyme), As refers to the absorbance of the sample (enzyme and inhibitor), and Asb is the absorbance of the sample blank (inhibitor without enzyme).

### 4.5. Statistical Analysis

Determination of α-glucosidase inhibitory activity was carried out in triplicate for each sample. The obtained results were presented as means ± standard deviation (SD), and were then compared using an unpaired *t*-test.

## 5. Conclusions

The utilized Zippertex technology of extraction with XAD-16 resin had offered the maximum qualitative and quantitative recovery of catechin, with minimum operations, handling, minimum solvent, and time in comparison with other used methods of extraction and isolation. Moreover, the α-glucosidase inhibitory activity of catechin isolated from the *A. unedo* roots exhibited a potential effect in comparison with commercially available α-glucosidase inhibitory agent acarbose which explains their traditional use as a hypoglycemic plant. These findings further imply that catechin or *A. unedo* root extract separated by Zippertex technology can be used as a potential antidiabetic agent.

## Figures and Tables

**Table 1:** The α-glucosidase inhibitory potency of catechin.

Compounds	α-Glucosidase Inhibitory Activity IC_50_ (μg/mL), ±SD
Catechin	87.55 ± 2.23
Acarbose	199.53 ± 1.12

## Supplementary Materials

The following are available online at http://www.mdpi.com/2223-7747/7/2/31/s1, Figure S1: ^1^H NMR spectra analysis, Figure S2: ^1^H NMR spectra analysis, Figure S3: ^1^H NMR spectra analysis, Figure S4: ^13^C NMR spectra analysis, Figure S5: ^13^C NMR spectra analysis, Figure S6: ^13^C NMR spectra analysis.

## References

[1] Wild S., Roglic G., Green A., Sicree R., King H. (2004). Global prevalence of diabetes: Estimates for the year 2000 and projections for 2030. Diabetes Care.

[2] American Diabetes Association (2017). Standards of medical care in diabetes-2017 abridged for primary care providers. Clin. Diabetes.

[3] Crawford K. (2017). Review of 2017 diabetes standards of care. Nurs. Clin. N. Am..

[4] Mazzotti A., Caletti M.T., Marchignoli F., Forlani G., Marchesini G. (2017). Which treatment for type 2 diabetes associated with non-alcoholic fatty liver disease?. Dig. Liver Dis..

[5] Eddouks M., Ouahidi M., Farid O., Moufid A., Khalidi A., Lemhadri A. (2007). L’utilisation des plantes médicinales dans le traitement du diabète au maroc. Phytothérapie.

[6] Bimakr M., Rahman R.A., Taip F.S., Ganjloo A., Salleh L.M., Selamat J., Hamid A., Zaidul I. (2011). Comparison of different extraction methods for the extraction of major bioactive flavonoid compounds from spearmint (*Mentha spicata* L.) leaves. Food Bioprod. Process..

[7] Nothias L.-F.L., Boutet-Mercey S.P., Cachet X., De La Torre E., Laboureur L., Gallard J.-F.O., Retailleau P., Brunelle A., Dorrestein P.C., Costa J. (2017). Environmentally friendly procedure based on supercritical fluid chromatography and tandem mass spectrometry molecular networking for the discovery of potent antiviral compounds from *Euphorbia semiperfoliata*. J. Nat. Prod..

[8] Barrajón-Catalán E., Herranz-López M., Joven J., Segura-Carretero A., Alonso-Villaverde C., Menéndez J.A., Micol V. (2014). Molecular promiscuity of plant polyphenols in the management of age-related diseases: Far beyond their antioxidant properties. Oxidative Stress and Inflammation in Non-Communicable Diseases-Molecular Mechanisms and Perspectives in Therapeutics.

[9] Mrabti H.N., Sayah K., Jaradat N., Kichou F., Ed-Dra A., Belarj B., Cherrah Y., Faouzi M.E.A. (2018). Antidiabetic and protective effects of the aqueous extract of *Arbutus unedo* L. in streptozotocin-nicotinamide-induced diabetic mice. J. Complement. Integr. Med..

[10] Mrabti H.N., Marmouzi I., Sayah K., Chemlal L., El Ouadi Y., Elmsellem H., Cherrah Y., Faouzi M.A. (2017). *Arbutus unedo* L. aqueous extract is associated with in vitro and in vivo antioxidant activity. J. Mater. Environ. Sci..

[11] Bento I., Pereira J.A. (2011). *Arbutus unedo* L. and its benefits on human health. J. Food Nutr. Res..

[12] Bnouham M., Merhfour F.Z., Ziyyat A., Aziz M., Legssyer A., Mekhfi H. (2010). Antidiabetic effect of some medicinal plants of oriental morocco in neonatal non-insulin-dependent diabetes mellitus rats. Hum. Exp. Toxicol..

[13] Pallauf K., Rivas-Gonzalo J., Del Castillo M., Cano M., de Pascual-Teresa S. (2008). Characterization of the antioxidant composition of strawberry tree (*Arbutus unedo* L.) fruits. J. Food Compos. Anal..

[14] Ruiz-Rodríguez B.-M., Morales P., Fernández-Ruiz V., Sánchez-Mata M.-C., Cámara M., Díez-Marqués C., Pardo-de-Santayana M., Molina M., Tardío J. (2011). Valorization of wild strawberry-tree fruits (*Arbutus unedo* L.) through nutritional assessment and natural production data. Food Res. Int..

[15] Mariotto S., Esposito E., Di Paola R., Ciampa A., Mazzon E., de Prati A.C., Darra E., Vincenzi S., Cucinotta G., Caminiti R. (2008). Protective effect of *Arbutus unedo* aqueous extract in carrageenan-induced lung inflammation in mice. Pharmacol. Res..

[16] Afkir S., Nguelefack T.B., Aziz M. (2008). *Arbutus unedo* prevents cardiovascular and morphological alterations in l-name-induced hypertensive rats: Part i: Cardiovascular and renal hemodynamic effects of *Arbutus unedo* in L-name-induced hypertensive rats. J. Ethnopharmacol..

[17] Novais M., Santos I., Mendes S., Pinto-Gomes C. (2004). Studies on pharmaceutical ethnobotany in Arrabida Natural Park (Portugal). J. Ethnopharmacol..

[18] Kivçak B., Mert T., Denizci A. (2001). Antimicrobial activity of *Arbutus unedo* L.. J. Pharm. Sci..

[19] Arias M., Penichet I., Ysambertt F., Bauza R., Zougagh M., Ríos Á. (2009). Fast supercritical fluid extraction of low-and high-density polyethylene additives: Comparison with conventional reflux and automatic soxhlet extraction. J. Supercrit. Fluids.

[20] Luthria D.L. (2008). Influence of experimental conditions on the extraction of phenolic compounds from parsley (*Petroselinum crispum*) flakes using a pressurized liquid extractor. Food Chem..

[21] Albuquerque B.R., Prieto M., Barreiro M.F., Rodrigues A., Curran T.P., Barros L., Ferreira I.C. (2017). Catechin-based extract optimization obtained from *Arbutus unedo* L. Fruits using maceration/microwave/ultrasound extraction techniques. Ind. Crop. Prod..

[22] Junior O.V., Dantas J.H., Barão C.E., Zanoelo E.F., Cardozo-Filho L., de Moraes F.F. (2017). Formation of inclusion compounds of (+) catechin with β-cyclodextrin in different complexation media: Spectral, thermal and antioxidant properties. J. Supercrit. Fluids.

[23] Matsui T., Tanaka T., Tamura S., Toshima A., Tamaya K., Miyata Y., Tanaka K., Matsumoto K. (2007). α-glucosidase inhibitory profile of catechins and theaflavins. J. Agric. Food Chem..

[24] Bhandari M.R., Jong-Anurakkun N., Hong G., Kawabata J. (2008). α-glucosidase and α-amylase inhibitory activities of nepalese medicinal herb pakhanbhed (*Bergenia ciliata*, haw.). Food Chem..

[25] Tadera K., Minami Y., Takamatsu K., Matsuoka T. (2006). Inhibition of α-glucosidase and α-amylase by flavonoids. J. Nutr. Sci. Vitaminol..

[26] Justino A.B., Miranda N.C., Franco R.R., Martins M.M., da Silva N.M., Espindola F.S. (2018). *Annona muricata* Linn. leaf as a source of antioxidant compounds with in vitro antidiabetic and inhibitory potential against α-amylase, α-glucosidase, lipase, non-enzymatic glycation and lipid peroxidation. Biomed. Pharmacother..

[27] Giovannini D., Gismondi A., Basso A., Canuti L., Braglia R., Canini A., Mariani F., Cappelli G. (2016). *Lavandula angustifolia* Mill. Essential oil exerts antibacterial and anti-inflammatory effect in macrophage mediated immune response to *Staphylococcus aureus*. Immunol. Investig..

[28] Ettorre A., Frosali S., Andreassi M., Di Stefano A. (2010). Lycopene phytocomplex, but not pure lycopene, is able to trigger apoptosis and improve the efficacy of photodynamic therapy in HL60 human leukemia cells. Exp. Biol. Med..

[29] Higdon J.V., Frei B. (2010). Tea catechins and polyphenols: Health effects, metabolism, and antioxidant functions. Crit. Rev. Food Sci. Nutr..

[30] Malongane F., McGaw L.J., Mudau F.N. (2017). The synergistic potential of various teas, herbs and therapeutic drugs in health improvement: A review. J. Sci. Food Agric..

[31] Selga A., Torres J.L. (2005). Efficient preparation of catechin thio conjugates by one step extraction/depolymerization of pine (*Pinus pinaster*) bark procyanidins. J. Agric. Food Chem..

[32] Rabhi C., Arcile G., Cariel L., Lenoir C., Bignon J., Wdzieczak-Bakala J., Ouazzani J. (2015). Antiangiogenic-like properties of fermented extracts of ayurvedic medicinal plants. J. Med. Food.

[33] Fichtali I., Laaboudi W., Hadrami E.E., Aroussi F.E., Ben-Tama A., Benlemlih M., Stiriba S. (2016). Synthesis, characterization and antimicrobial activity of novel benzophenone derived 1, 2, 3-triazoles. J. Mater. Environ. Sci..

[34] Kee K.T., Koh M., Oong L.X., Ng K. (2013). Screening culinary herbs for antioxidant and α-glucosidase inhibitory activities. Int. J. Food Sci. Technol..

